# Neutrophil Extracellular Traps Contain Selected Antigens of Anti-Neutrophil Cytoplasmic Antibodies

**DOI:** 10.3389/fimmu.2017.00439

**Published:** 2017-04-13

**Authors:** Rachita Panda, Thorsten Krieger, Luke Hopf, Thomas Renné, Friedrich Haag, Nadja Röber, Karsten Conrad, Elena Csernok, Tobias A. Fuchs

**Affiliations:** ^1^Laboratory of Molecular Inflammation, Institute of Clinical Chemistry and Laboratory Medicine, University Medical Center Hamburg-Eppendorf, Hamburg, Germany; ^2^Diagnostic Center, Institute of Clinical Chemistry and Laboratory Medicine, University Medical Center Hamburg-Eppendorf, Hamburg, Germany; ^3^Department of Molecular Medicine and Surgery, Karolinska Institute, Solna, Sweden; ^4^Institute of Immunology, University Medical Center Hamburg-Eppendorf, Hamburg, Germany; ^5^Institute of Immunology, Technical University Dresden, Dresden, Germany; ^6^Vasculitis-Center Tübingen-Kirchheim, Department of Internal Medicine, Rheumatology and Immunology, University Hospital Kirchheim, Kirchheim, Germany

**Keywords:** neutrophil extracellular traps, NETosis, anti-neutrophil cytoplasmic antibodies, P-ANCA, C-ANCA, ANCA-associated vasculitis

## Abstract

Neutrophil extracellular traps (NETs) are chromatin filaments decorated with enzymes from neutrophil cytoplasmic granules. Anti-neutrophil cytoplasmic antibodies (ANCAs) bind to enzymes from neutrophil cytoplasmic granules and are biomarkers for the diagnosis of systemic vasculitides. ANCA diagnostics are based on indirect immunofluorescence (IIF) of ethanol-fixed neutrophils. IIF shows a cytoplasmic staining pattern (C-ANCA) due to autoantibodies against proteinase 3 (PR3) or a perinuclear staining pattern (P-ANCA) due to autoantibodies against myeloperoxidase (MPO). The distinct ANCA-staining patterns are an artifact of ethanol fixation. Here, we tested NETs as a substrate for the detection of ANCAs in human sera. We observed that P-ANCAs specifically stained NETs, while C-ANCAs targeted the cell bodies of netting neutrophils. The distinct ANCA-staining patterns were caused by the presence of MPO, but not PR3, in NETs. Using NETs as a substrate for IIF, we characterized ANCAs in sera of patients with ANCA-associated vasculitis (AAV). Furthermore, we inhibited serine proteases by diisopropylfluorophosphate to prevent chromatin unfolding and the release of NETs and thus generated neutrophils with MPO-positive nuclei and PR3-positive cytoplasm, which resembled the appearance of ethanol-fixed neutrophils. In conclusion, our data suggest that NETs are selectively loaded with antigens recognized by P-ANCAs, and netting neutrophils provide a physiological substrate for ANCA detection in patients with AAV.

## Introduction

Neutrophil extracellular traps (NETs) are lattices of intact DNA filaments containing histones and neutrophil enzymes (1). NETs are released by activated neutrophils and display diverse functions in infections, sterile inflammation, and autoimmune diseases (2). Neutrophils form NETs by a multi-step cell death program (NETosis), which involves the breakdown of cytoplasmic granules and the nuclear envelope (3, 4). The loss of internal membranes enables the intracellular loading of chromatin filaments with enzymes from neutrophil cytoplasmic granules (3, 4).

Anti-neutrophil cytoplasmic antibodies (ANCAs) bind to enzymes from neutrophil cytoplasmic granules (5–7). ANCAs are associated with vasculitis, glomerulonephritis, and several autoimmune diseases of the gastrointestinal tract (8–10). The detection of ANCAs is based on the screening of patient sera by indirect immunofluorescence (IIF) using ethanol-fixed unstimulated neutrophils as a substrate (7). IIF identifies two main types of ANCAs: a cytoplasmic staining pattern indicates C-ANCAs with specificity for proteinase 3 (PR3) (6, 11), whereas a perinuclear staining pattern indicates P-ANCAs mainly with a specificity for myeloperoxidase (MPO) (5). PR3- and MPO-ANCAs are diagnostic markers for two types of ANCA-associated vasculitis (AAV), granulomatosis with polyangiitis (GPA) (9) and microscopic polyangiitis (MPA) (5), respectively. NETs have been reported to contain enzymes from all types of neutrophil granules including MPO and PR3 (12, 13). We therefore hypothesized that NETs are a substrate for the detection of ANCAs in patient sera.

## Materials and Methods

### Neutrophil Isolation

Neutrophils were isolated as previously described (3). Peripheral blood was collected from healthy volunteer donors and anticoagulated with ethylenediaminetetraacetic acid (EDTA, monovette, Sarstedt). The ethics committee of University Medical Center Hamburg-Eppendorf approved the blood collection from volunteer donors (PV 4616). Blood was layered onto Histopaque 1119 (Sigma-Aldrich). After centrifugation for 20 min at 800 *g*, the neutrophil-rich layer was collected. The cells were washed with Hanks-buffered salt solution without divalent cations (HBSS−, Life Technologies) supplemented with 5 mM EDTA and 0.1% bovine serum albumin (BSA, Sigma-Aldrich). Washed cells were further fractionated on a discontinuous Percoll gradient (GE Healthcare). After centrifugation for 20 min at 800 *g*, the neutrophil-rich layer was collected and washed with 0.1% BSA in HBSS−. All procedures were conducted at room temperature. Neutrophil viability was greater than 98%, as determined by trypan blue (Sigma-Aldrich) exclusion.

### Induction of NETosis

Isolated neutrophils were seeded into sterile 96 well optical plates (Falcon) coated with 0.001% poly-l-lysine (Sigma-Aldrich) at a concentration of 5 × 10^4^ cells per well in Dulbecco’s modified Eagle medium (Life Technologies). For non-activated cells, the naïve neutrophils were seeded into the plate and incubated for 15 min to allow the adherence of cells to the wells. To induce NET formation, 0.1 µM phorbol 12-myristate 13-acetate (PMA, Sigma-Aldrich) was added, and the cells were then incubated for 4 h. In selected experiments, diisopropylfluorophosphate (DFP, Sigma-Aldrich) was added at 20 mM or at indicated concentrations to the cells 5 min before addition of 0.1 µM PMA, and the cells were incubated for up to 15 h. All incubations were performed at 37°C with 5% CO_2_. The cells were fixed with 2% paraformaldehyde (PFA, Sigma-Aldrich) overnight at 4°C in case of non-activated cells and NETs. NETotic cells were fixed for 2 h at 4°C.

### Indirect Immunofluorescence

Prior to immunostaining, PFA-fixed neutrophils were washed twice with phosphate-buffered saline (PBS). The cells were permeabilized with 0.5% Triton X-100 diluted in PBS for 5 min. For blocking, the wells were incubated with 2% BSA in PBS. Primary and secondary antibodies were used at the following concentrations: 5 µg/ml mouse antihuman MPO antibody (Hycult Biotech, Clone 266-6K1, product number: HM2164), 5 µg/ml of mouse antihuman PR3 antibody (Clone WGM2) (14), 2.5 µg/ml rabbit antihuman MPO antibody (DAKO, product number: A0398), 5 µg/ml rabbit antihuman lactoferrin (LF) antibody (Sigma-Aldrich, product number: L3262), 5 µg/ml mouse antihuman neutrophil elastase (NE) (DAKO, Clone NP57, product number: M0752), 5 µg/ml of Alexa Fluor-546 goat anti-rabbit IgG, 10 µg/ml of Alexa Fluor-488 goat anti-mouse IgG, and 10 µg/ml Alexa Fluor-555 goat-antihuman IgG (all Life Technologies). Primary antibodies were diluted in PBS with 0.05% Tween (PBST). Secondary antibodies were diluted in PBST with 2% BSA. Primary and secondary antibodies were incubated with cells at 37°C for 30 and 45 min, respectively. The wells were washed twice with PBST for 5 min before the addition of the secondary antibodies. DNA was stained with 5 µg/ml 4′,6-diamidino-2-phenylindole (Sigma-Aldrich). Fluorescent images were acquired on the day of the IIF staining using a confocal microscope (Leica TCS SP8) or if not stated otherwise with an inverted fluorescence microscope (Zeiss Axiovert 200 M). Images were analyzed using ImageJ software (NIH).

### Commercial ANCA-Detection Kit and ANCA Sera

We used commercially available P-ANCA-positive serum (Euroimmun, product number: CA 1211-0105-3, titer 1/32), C-ANCA-positive serum (Euroimmun, product number: CA1200-0110-3, titer 1/32), and autoantibody-free serum (Euroimmun, product number: CA1000-0110). For Figures 7 and 8, we used a commercial kit with ethanol- and formalin-fixed neutrophils to detect neutrophil granular enzymes and ANCA by IIF (Euroimmun, product number: FA1201-1005-22). Sera were diluted 20-fold in PBST supplemented with 5 mM EDTA and 0.1% BSA before the analysis by IIF.

### Patient Sera

Sera from patients with GPA and MPA were diluted 40-fold in PBST supplemented with 5 mM EDTA and 0.1% BSA before the analysis by IIF. The ethics committee of Medical Faculty of the Technical University Dresden approved the use of the patient sera for this study (EK 226112006).

### Statistical Evaluation

Statistical analysis was performed using Prism Software (GraphPad, USA) and one-way-ANOVA followed by Tukey’s multiple comparison test. Results were considered significant at *p* < 0.05.

## Results

### P-ANCAs Target NETs, and C-ANCAs Bind to Cell Bodies of Netting Neutrophils

**Figure 1:**
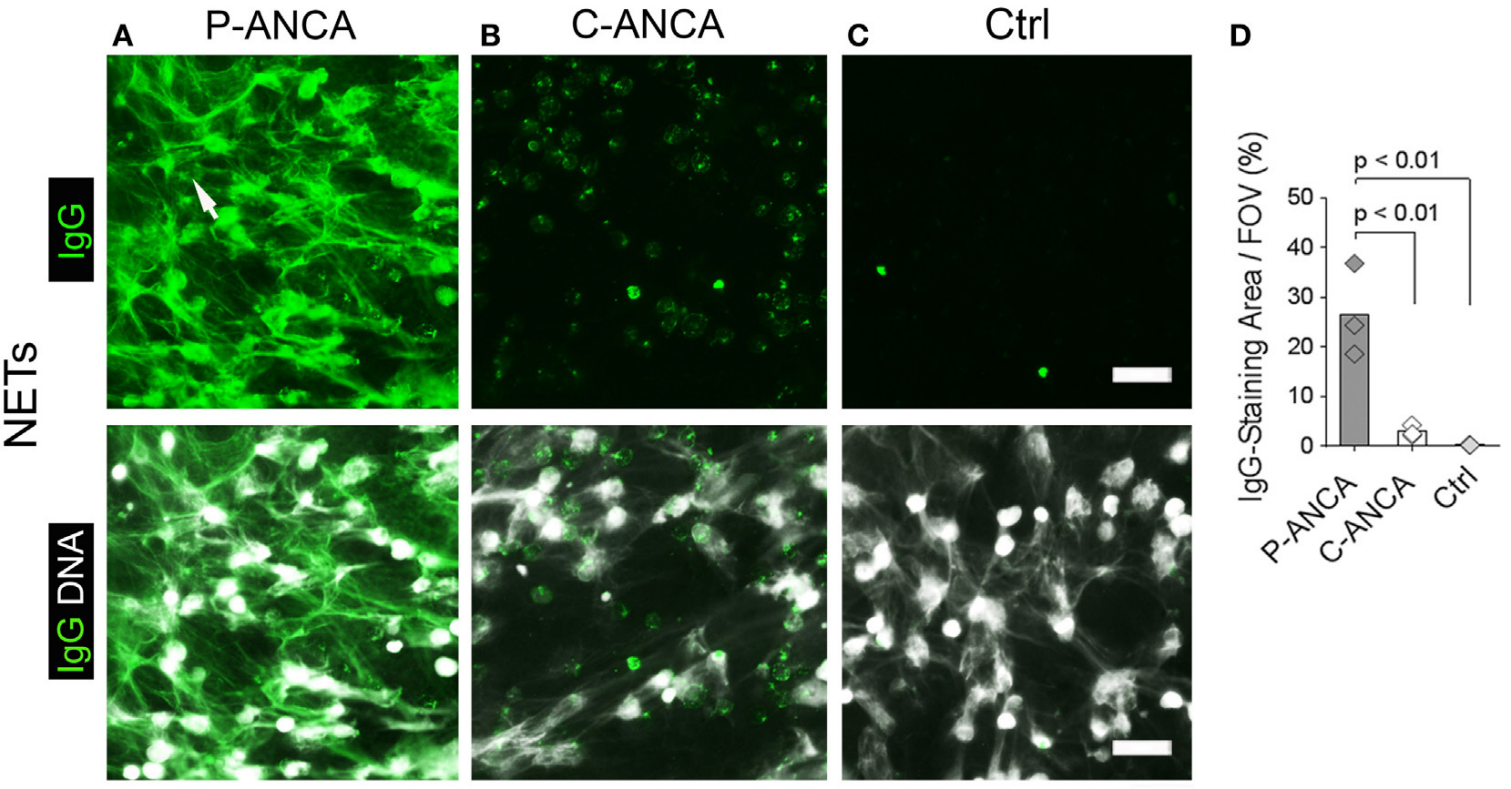
**P-ANCAs target neutrophil extracellular traps (NETs), C-ANCAs bind to cell bodies of netting neutrophils**. **(A–C)** Stainings of phorbol 12-myristate 13-acetate (PMA)-activated neutrophils. Top panel shows human IgG staining (green), and bottom panel shows overlay of human IgG (green) and DNA staining (white). Bar represents 50 µm. **(A)** Staining of activated neutrophils incubated with P-ANCA-positive sera shows NETs (arrow). **(B)** C-ANCA-positive sera do not stain NETs, but the cell bodies of netting neutrophils. **(C)** Staining with autoantibody-free sera (Ctrl) does not stain neutrophils or NETs. **(D)** Quantification of the IgG-staining area per field of view (FOV) of PMA-activated neutrophils incubated with P-ANCAs (*n* = 3), C-ANCAs (*n* = 3), or Ctrl sera (*n* = 3). *p*-Values were calculated using one-way-ANOVA followed by Tukey’s multiple comparison test.

To test whether ANCAs bind to NETs, we induced NETosis by activating human neutrophils for 4 h with PMA, a potent inducer of NETs (1, 3). DNA staining showed NETs as abundant lattices of extracellular DNA filaments covering the intercellular space between activated neutrophils (Figures 1A–C). Incubation of NETs with ANCA-positive sera revealed that P-ANCAs prominently and specifically stained NETs (Figure 1A). C-ANCA-positive sera did not bind to NETs but targeted the cell bodies of netting neutrophils (Figure 1B). As negative control, we used sera without detectable autoantibodies, which stained neither NETs nor netting neutrophils (Figure 1C). NETs exceed the size of netting neutrophils by several magnitudes (1). Consequently, quantification of human IgGs showed that P-ANCAs, but not C-ANCAs stained large areas of PMA-activated neutrophils (Figure 1D).

**Figure 2:**
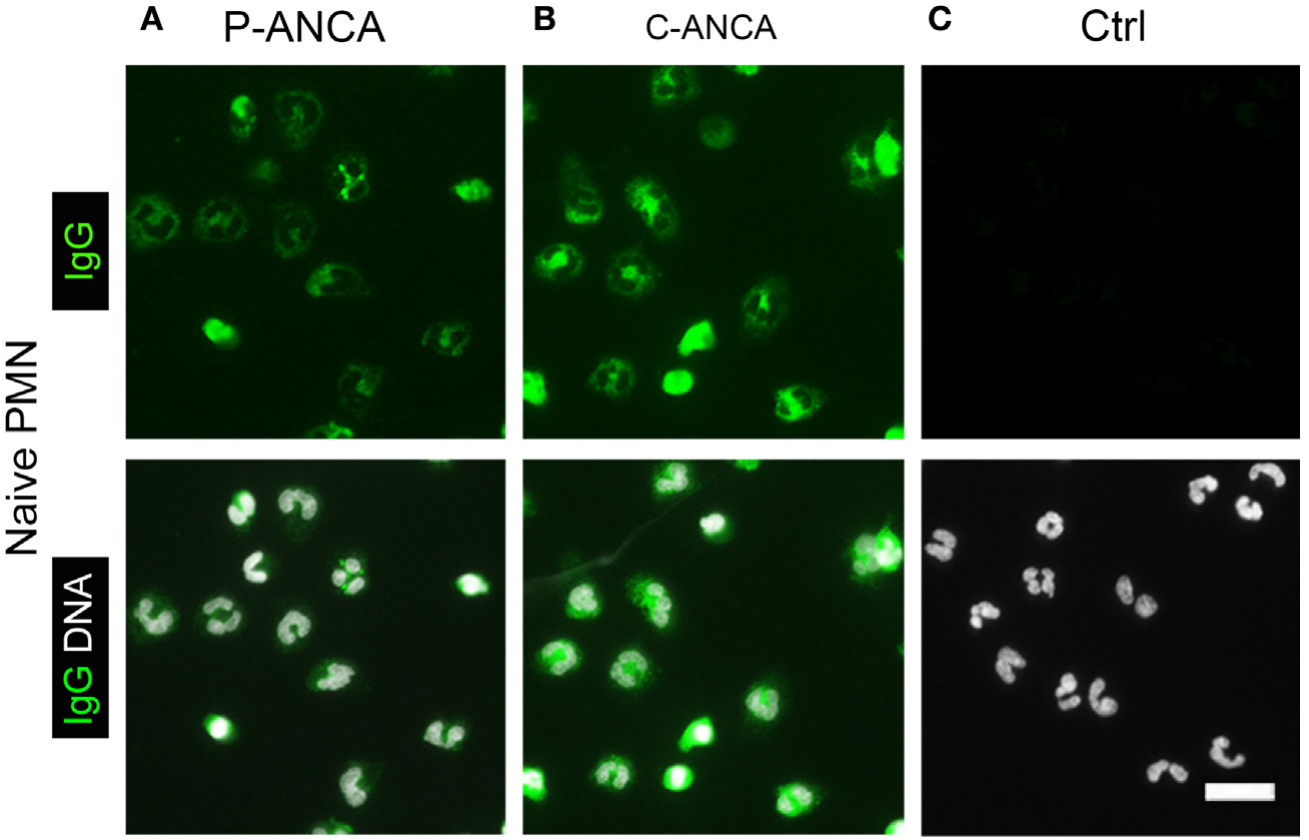
**P-ANCAs and C-ANCAs stain the cytoplasm of unstimulated neutrophils**. **(A–C)** Staining of naïve neutrophils. Top panel shows human IgG staining (green), and bottom panel shows overlay of human IgG (green) and DNA staining (white). DNA staining of unstimulated neutrophils shows lobulated nuclei. Bar represents 20 µm. **(A)** Naïve neutrophils incubated with P-ANCA-positive sera and **(B)** C-ANCA-positive sera give a cytoplasmic staining pattern. **(C)** No staining was observed with autoantibody-negative sera (Ctrl). Images are representative of at least three independent experiments.

**Figure 3:**
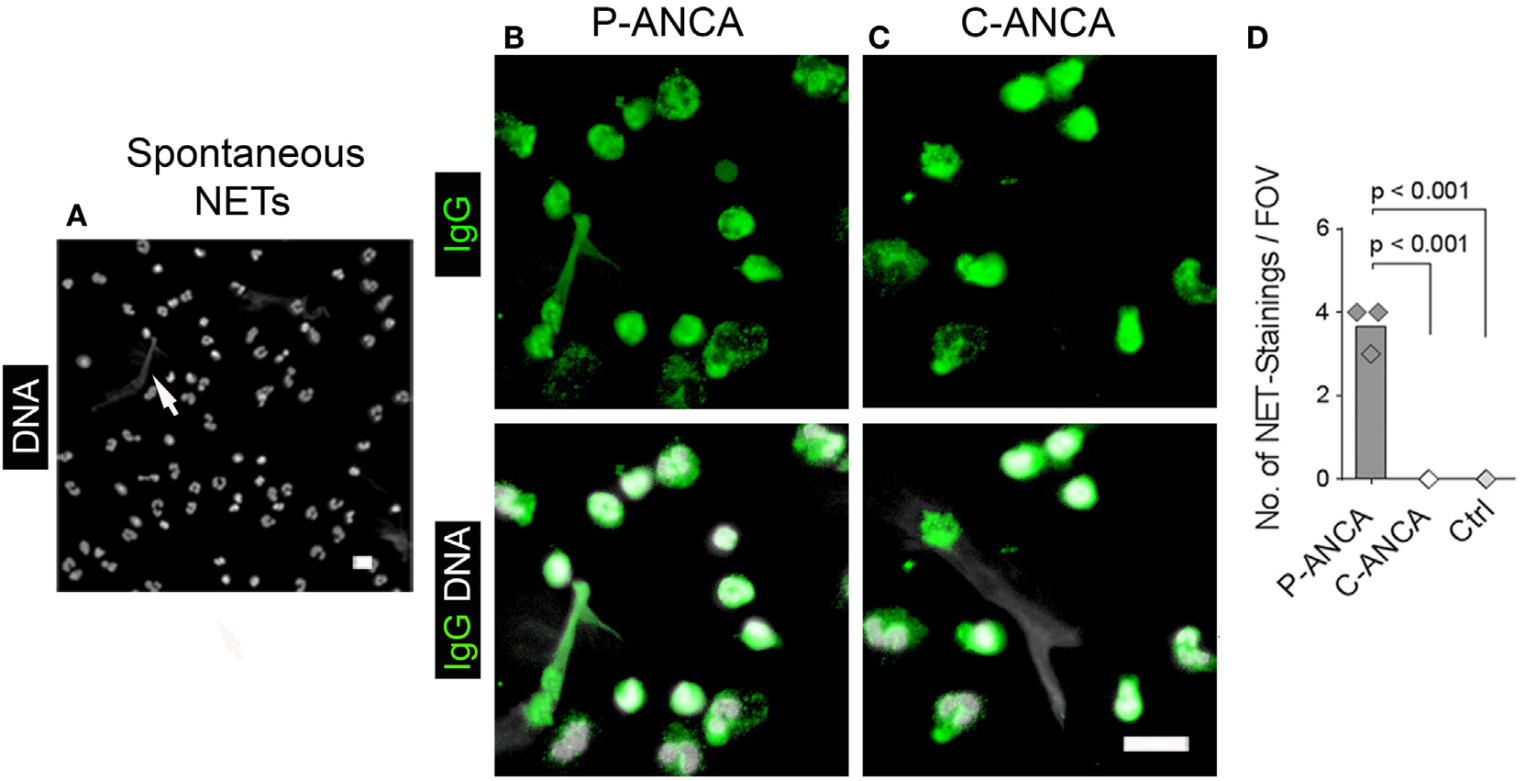
**P-ANCAs but not C-ANCAs bind to spontaneously formed neutrophil extracellular traps (NETs)**. **(A–C)** Staining of spontaneously formed NETs. Bar represents 20 µm. **(A)** DNA staining (white) shows nuclei of neutrophils and spontaneously formed NETs (arrow). Bar represents 20 µm. **(B,C)** Top panel shows human IgG staining (green), and bottom panel shows overlay of human IgG (green) and DNA staining (white). **(B)** P-ANCA positive sera stained a spontaneously formed NET shown in panel A along with the cytoplasm of naïve cells. **(C)** C-ANCA positive sera stained the cytoplasm of naïve cells only. Bar represents 20 µm. **(D)** Quantification of the number of NET stainings per field of view (FOV) in naïve neutrophils incubated with P-ANCAs (*n* = 3), C-ANCAs (*n* = 3), or autoantibody-negative sera (Ctrl, *n* = 3). *p*-Values were calculated using one-way-ANOVA followed by Tukey’s multiple comparison test.

We used naïve, non-activated neutrophils to confirm the specificity of the tested ANCA sera for targets in neutrophil cytoplasmic granules. DNA staining of unstimulated neutrophils showed the characteristic lobulated nuclei (Figures 2A–C). Both P- and C-ANCA stained granules in the cytoplasm, but not the nucleus (Figures 2A,B). Autoantibody-negative sera did not stain neutrophils (Figure 2C). To exclude that the specificity of P-ANCAs for NETs is restricted to PMA-induced NETs, we analyzed neutrophils, which formed NETs spontaneously. Short-term incubation of freshly isolated neutrophils in serum-free conditions induced the formation of NETs in a few cells spontaneously, while the vast majority of neutrophils retain their lobulated-shaped nucleus (Figure 3A) (1, 3). P-ANCA-positive sera stained the spontaneously formed NETs as well as the cytoplasm of naïve neutrophils (Figure 3B), whereas C-ANCA-positive sera stained only naïve neutrophils (Figure 3C). Quantification of the stainings of spontaneously formed NETs confirmed the specificity of P-ANCA-positive sera for NETs (Figure 3D). In conclusion, these data show that NETs contain antigens targeted by P-ANCAs, but not C-ANCAs.

### NETs Are a Substrate to Discriminate Sera from Patients with P- and C-ANCAs

**Figure 4:**
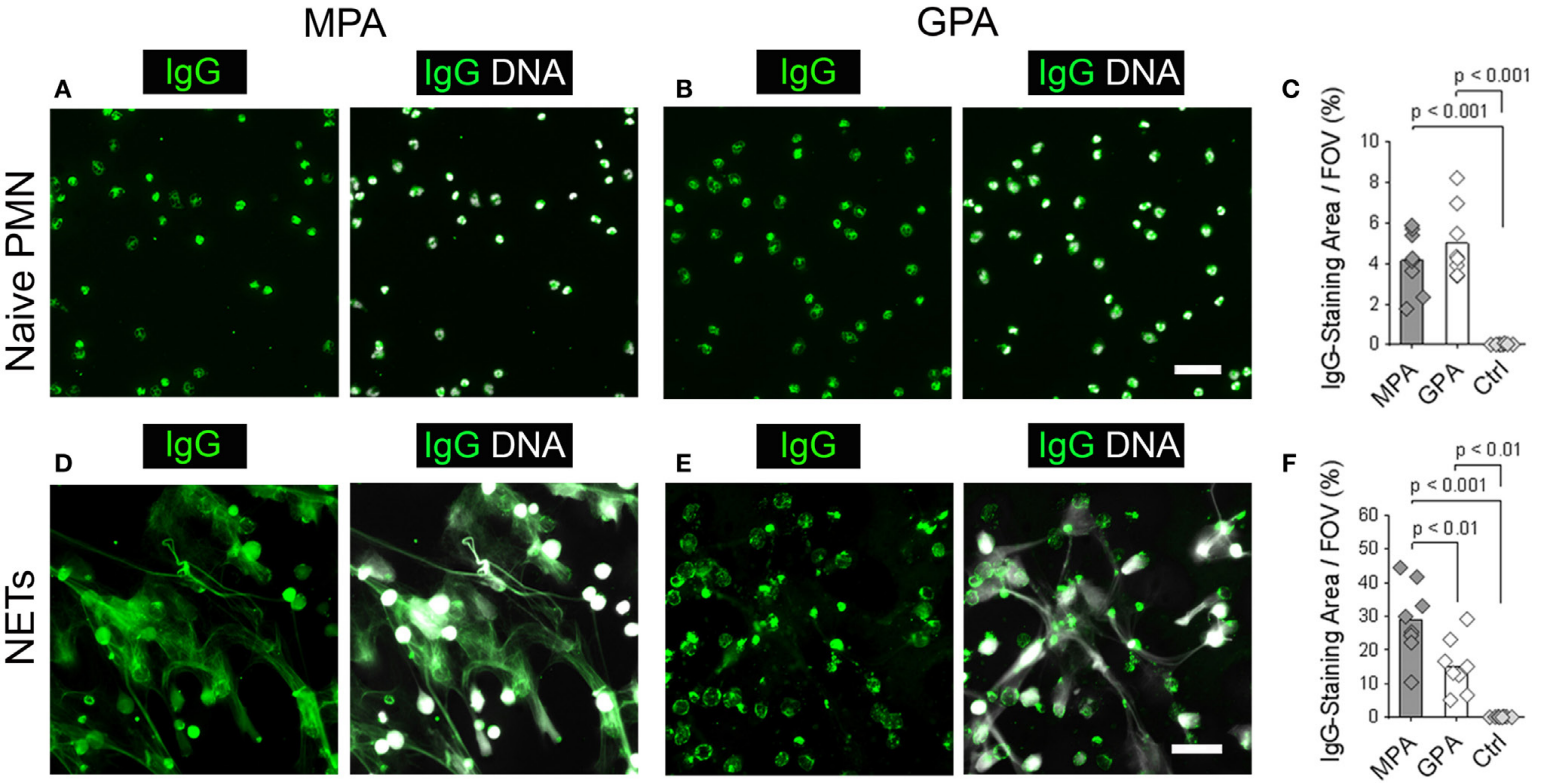
**Neutrophil extracellular traps (NETs) are a substrate to discriminate sera from microscopic polyangiitis (MPA) and granulomatosis with polyangiitis (GPA) patients**. **(A–C)** Naive neutrophils and **(D–F)** NETs form by phorbol 12-myristate 13-acetate-activated neutrophils were incubated with sera from patients with MPA (*n* = 8), patients with GPA (*n* = 8), and sera from healthy controls (Ctrl; *n* = 8). Left panel shows human IgG staining (green), and right panel shows overlay of human IgG (green) and DNA staining (white). Scale bars represent 50 µm. **(A)** Sera from MPA patients and **(B)** GPA patients show a cytoplasmic staining pattern in naïve neutrophils. **(C)** Quantification of the IgG-staining area per field of view (FOV) of naïve neutrophils. **(D)** Sera from MPA patients show a web-like staining pattern indicating the staining of NETs. **(E)** Sera from GPA patients show reactivity with the cell bodies of netting neutrophils. **(F)** Quantification of the IgG-staining area per FOV of NETs. *p*-Values in panels **(C,F)** were calculated using one-way-ANOVA followed by Tukey’s multiple comparison test.

These results suggest that NETs may serve as an IIF substrate to discriminate patients with P-ANCAs from patients with C-ANCAs. To test this hypothesis, we analyzed sera from eight MPA patients and eight GPA patients. Eight sera from healthy donors were included as negative controls. We chose MPA and GPA sera, which stained ethanol-fixed neutrophils, the conventional substrate for ANCA detection, with a similar intensity (IIF titers, MPA: 420 ± 190.03, GPA: 640 ± 418.97, *p* > 0.05). We analyzed the patient and control sera by IIF using unstimulated naïve neutrophils and NETs as a substrate. Sera from MPA and GPA patients showed a similar cytoplasmic staining pattern on unstimulated neutrophils (Figures 4A,B). Consequently, autoreactive IgGs in MPA and GPA sera stained only the small area of the neutrophil cell bodies (Figure 4C). Control sera from healthy donors did not stain neutrophils (Figure 4C). Using NETs from PMA-activated neutrophils as a substrate, we observed a web-like staining pattern in the intercellular space with sera from MPA patients (Figure 4D), whereas sera from GPA patients stained the cell bodies of netting neutrophils (Figure 4E). In line with these results, quantification revealed larger stainings by autoantibodies in sera from MPA patients compared to GPA patients (Figure 4F). Control sera did not stain NETs or netting neutrophils (Figure 4F). In summary, IIF staining of NETs allows the discrimination of P- and C-ANCA in sera from patients with ANCA-associated vasculitis.

### NETs Contain Selected Enzymes from Neutrophil Cytoplasmic Granules

**Figure 5:**
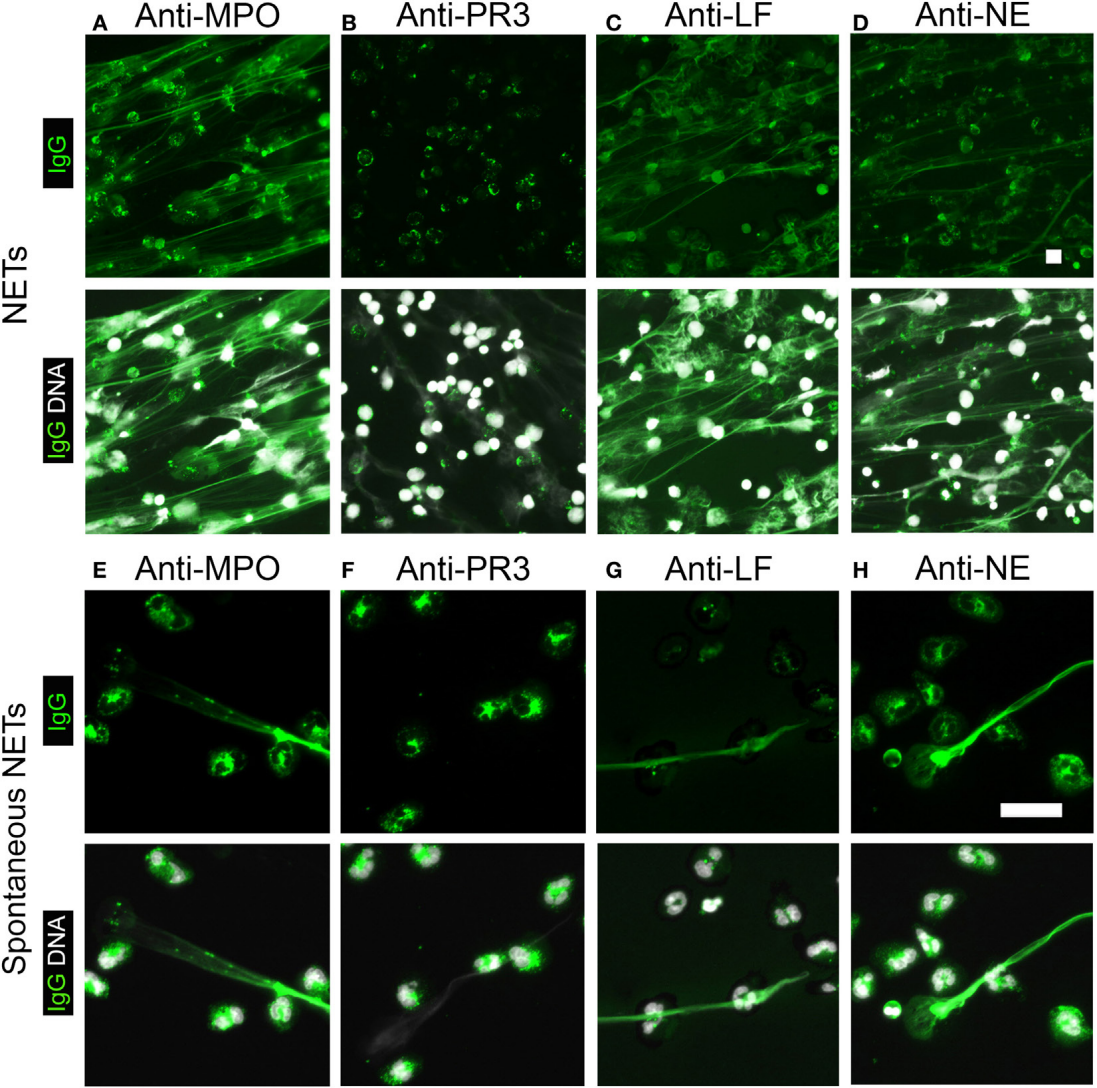
**Neutrophil extracellular traps (NETs) contain selected enzymes from neutrophil cytoplasmic granules**. Top panel shows human IgG staining (green), and bottom panel shows overlay of human IgG (green) and DNA staining (white). **(A–D)** Stainings of NETs from phorbol 12-myristate 13-acetate-activated neutrophils. DNA staining (white) shows NETs as elongated fibers. Bar represents 50 µm. **(A)** Staining with anti-myeloperoxidase (MPO)-IgG (green) shows NETs. **(B)** Anti-proteinase 3 (PR3)-antibodies do not stain NETs (green), but label cell bodies of netting neutrophils. **(C)** Anti-lactoferrin (LF) antibodies and **(D)** anti-neutrophil elastase (NE)-IgG stain NETs. **(E–H)** Staining of naïve neutrophils along with spontaneous NETs. DNA staining shows nuclei of neutrophils and spontaneously formed NETs. Bar represents 20 µm. **(E)** Anti-MPO-IgG stained spontaneously formed NET along with the cytoplasm of naïve cells. **(F)** Anti-PR3-IgG stained the cytoplasm of naïve cells but not spontaneously formed NET. **(G)** Anti-LF antibodies and **(H)** anti-NE antibodies stained the cytoplasm of naïve cells and spontaneously formed NET. Images are representative of at least three independent experiments.

Myeloperoxidase and PR3 are the predominant antigens recognized by P- and C-ANCAs, respectively (5, 11). We therefore speculated that NETs are rich in MPO, but not PR3. Indeed, staining of NETs from PMA-activated neutrophils with anti-MPO antibodies showed the co-localization of MPO and NET-DNA fibers (Figure 5A), whereas anti-PR3-antibodies targeted the cell bodies of netting neutrophils (Figure 5B). Additional known antigens of P-ANCAs are LF (15) and NE (16, 17). Staining with specific antibodies against LF and NE showed that both enzymes are present in NETs (Figures 5C,D), confirming previous reports (1). Using unstimulated neutrophils and spontaneously formed NETs as a substrate, IIF for antibodies against MPO, PR3, LF, and NE showed a cytoplasmic pattern in cells with lobulated nuclei (Figures 5E–H), whereas the spontaneously formed NETs stained only for MPO (Figure 5E), LF (Figure 5F), and NE (Figure 5G), but not for PR3 (Figure 5H). Control IgG did not stain neutrophils or NETs. Taking together, these data suggest that during NETosis, NETs are selectively loaded with enzymes targeted by P-ANCAs, while the C-ANCA antigen PR3 resides in the cell bodies of netting neutrophils.

### Serine Protease Inhibition in NETosis Generates Neutrophils with MPO-Positive Nuclei

**Figure 6:**
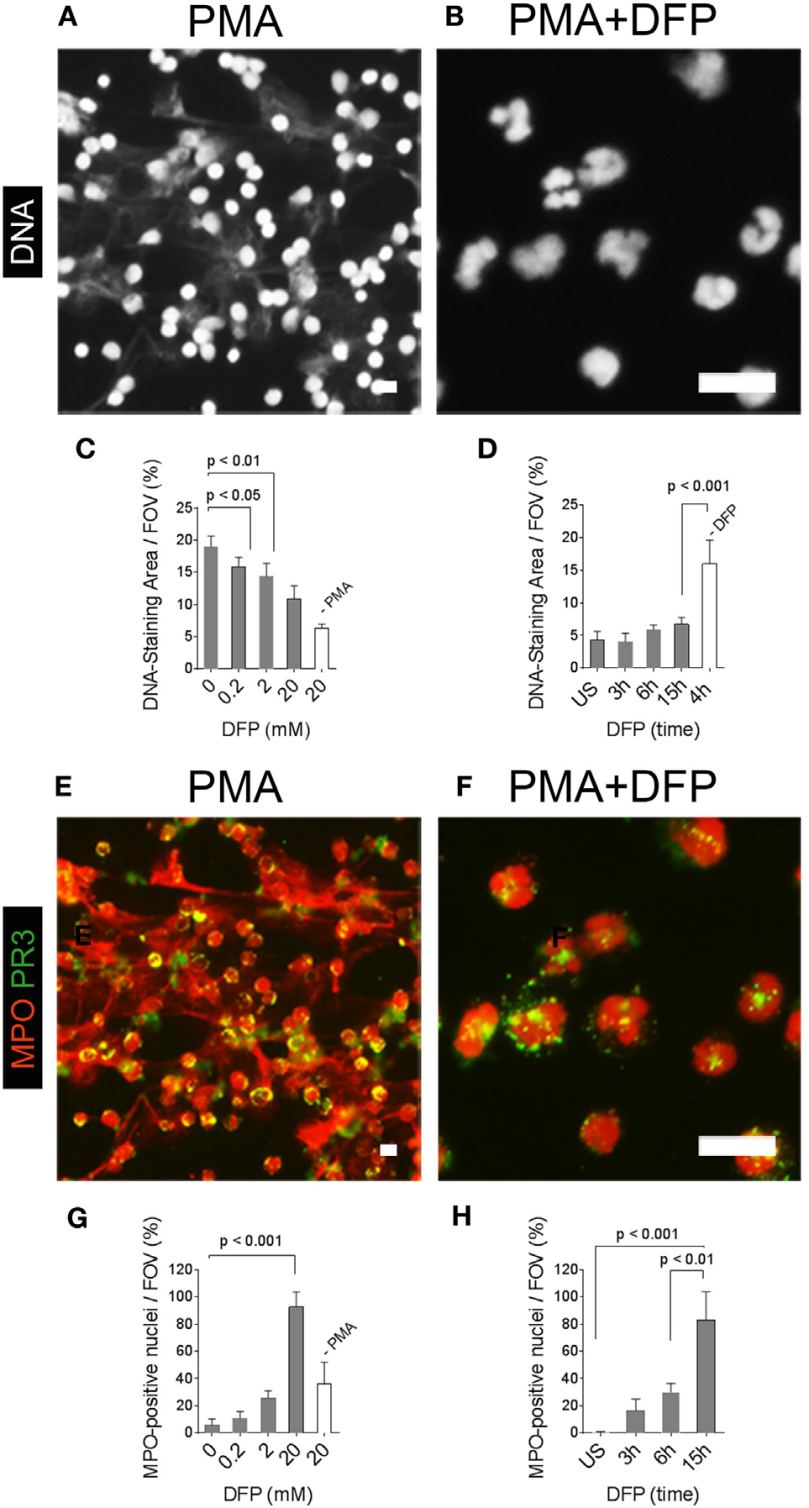
**Pharmacological inhibition of neutrophil extracellular trap (NET)-formation generates neutrophils with myeloperoxidase (MPO)-positive nuclei**. Neutrophils were activated with phorbol 12-myristate 13-acetate (PMA) in the absence or presence of the serine-protease inhibitor diisopropylfluorophosphate (DFP). **(A)** DNA staining (white) shows NETs in PMA-activated neutrophils. **(B)** DFP at a concentration of 20 mM blocked the generation of NETs and neutrophil nuclei retained their lobulated shape. Scale bars in panels **(A,B)** represent 50 µm. **(C)** Quantification of the DNA-staining area per field of view (FOV) of PMA-activated neutrophils and naïve neutrophils (−PMA) incubated for 15 h in the presence of indicated concentrations of DFP. **(D)** Quantification of the DNA-staining area per FOV of naïve neutrophils [unstimulated (US)] and neutrophils activated with PMA in the presence of 20 mM DFP for indicated time points. Control neutrophils were activated with PMA for 4 h in the absence of DFP (−DFP). **(E)** Staining for myeloperoxidase (MPO, red) and proteinase 3 (PR3, green) shows MPO in NETs and PR3 in the cell bodies of neutrophils activated with PMA in the absence of PMA. **(F)** DFP at 20 mM blocked the formation of NETs and generated neutrophils with MPO-positive nuclei and PR3 retained its cytoplasmic localization. **(G)** Quantification of neutrophils with MPO-positive nuclei per FOV of PMA-activated neutrophils and naïve neutrophils (−PMA) incubated for 15 h in the presence of indicated DFP concentrations. **(H)** Quantification of neutrophils with MPO-positive nuclei per FOV of naïve neutrophils (US) and PMA-activated neutrophils in the presence of 20 mM DFP for indicated time points. Scale bars in panels **(G,H)** represent 50 µm.

This conclusion is supported by a previous study, which showed that NE and MPO, but not PR3, translocate to the nucleus in the initial phases of NETosis (4). Within the nucleus, NE mediates the unfolding of chromatin by cleaving histones, and NE is therefore required for NET formation (4). We activated neutrophils with PMA in the presence of DFP, which inhibits the enzymatic activity of neutrophil serine proteases, including NE (6, 18). DFP blocked the generation of NETs and nuclei of PMA-activated neutrophils retained their lobulated shape (Figures 6A,B). Quantification of the area covered by DNA from PMA-activated neutrophils showed that the inhibition of NET formation by DFP was dose dependent (Figure 6C) and effective even at prolonged activation times (Figure 6D). Localization of MPO by immunostaining showed abundant MPO in NETs (Figure 6E), whereas MPO was localized to nucleus in neutrophils activated with PMA in the presence of DFP (Figure 6F). The nuclear translocation of MPO in the presence of DFP was dose and time dependent and detectable in vast majority of neutrophils (Figures 6G,H). DFP did not cause a nuclear translocation of PR3, which was detected within the cytoplasm even at the highest DFP concentration (Figure 6F). The data suggest that DFP inhibits the nuclear breakdown during NETosis, but not the translocation of neutrophil enzymes such as MPO into the nucleus. Therefore, activation in the presence of DFP generates neutrophils with characteristic morphological features of NETosis, which we termed “NETotic neutrophils.”

### Ethanol Fixation Mimics NETosis

**Figure 7:**
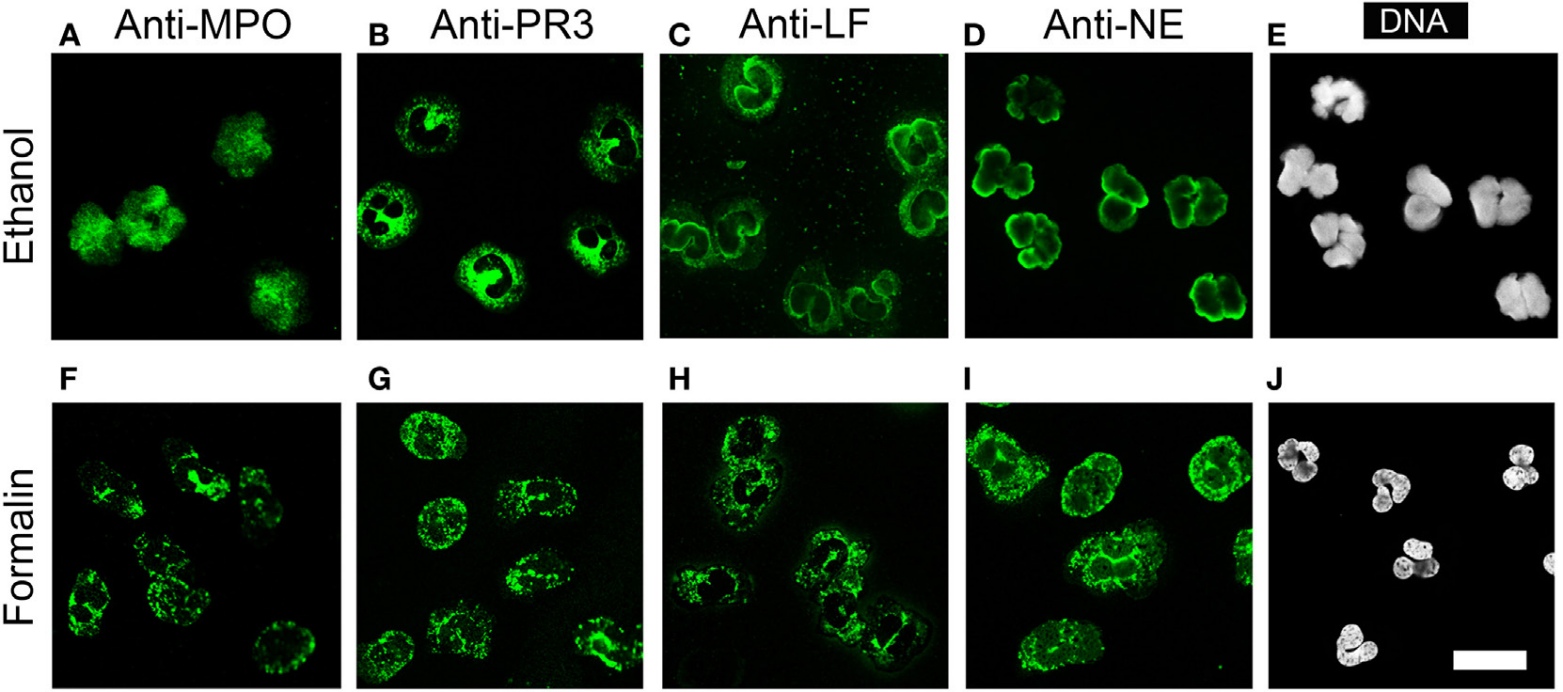
**Ethanol fixation of naïve neutrophils mimics NETosis**. **(A–D)** Staining of ethanol-fixed naïve neutrophils. **(A)** Anti-myeloperoxidase IgG (MPO, green) shows a nuclear staining. **(B)** Anti-proteinase 3 (PR3) IgG shows a cytoplasmic staining. **(C)** Anti-lactoferrin (LF) IgG and **(D)** anti-neutrophil elastase (NE) IgG shows peripheral nuclear staining. **(E)** DNA staining of ethanol-fixed neutrophils shows lobulated nuclei. **(F–I)** Staining on formalin fixed neutrophils with **(F)** anti-MPO IgG, **(G)** anti-PR3 IgG, **(H)** anti-LF IgG, and **(I)** anti-NE IgG shows a granular cytoplasmic staining pattern. **(J)** DNA staining of formalin-fixed neutrophils shows lobulated nuclei. All images were taken with a confocal microscope. Images are representative of at least 3 independent experiments. Bar represents 50 µm.

**Figure 8:**
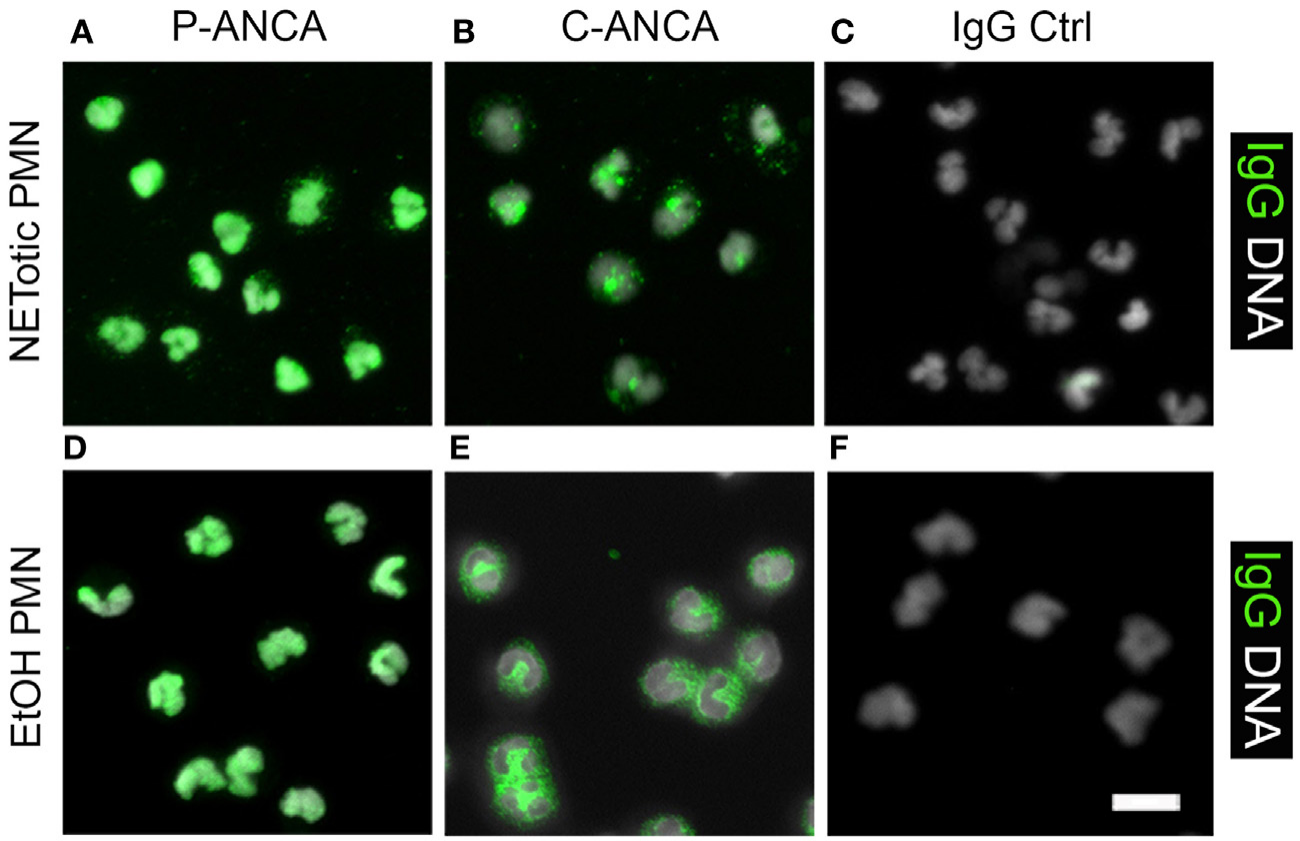
**Comparison of anti-neutrophil cytoplasmic antibody (ANCA) stainings on ethanol-fixed neutrophils and NETotic neutrophils**. Overlay of DNA staining (white) and human IgG staining (green). **(A–C)** Staining of NETotic neutrophils. **(A)** P-ANCA positive sera stain the nucleus of NETotic neutrophils. **(B)** C-ANCA positive sera stain the cytoplasm of NETotic cells. **(C)** Staining with autoantibody-free sera (IgG Ctrl) did not stain the cells. **(D–F)** Staining of ethanol-fixed naïve neutrophils with ANCA-positive sera. **(D)** P-ANCA positive sera stain the nucleus of ethanol-fixed naïve neutrophil. **(E)** C-ANCA positive sera stain the cytoplasm. **(F)** Staining with autoantibody-free sera (Ctrl) did not stain the cells. Images are representative of at least three independent experiments. Bar represents 50 µm.

NETotic neutrophils resemble the appearance of ethanol-fixed neutrophils, which are a commonly used and commercially available substrate for ANCA testing. Ethanol fixation of neutrophils causes the translocation of P-ANCA antigens from cytoplasmic granules toward the nucleus, while PR3 remains in the cytoplasm (7). Immunostaining of ethanol-fixed neutrophils showed a prominent nuclear staining of MPO (Figure 7A) and a staining in or at the periphery of the nucleus for LF (Figure 7C) and NE (Figure 7D). Staining for PR3 showed a cytoplasmic staining only (Figure 7B). Staining of unstimulated neutrophils fixed with formalin showed a cytoplasmic and granular staining pattern for all neutrophil enzymes tested (Figures 7F–I). In conclusion, these data illustrate that the fixation with ethanol selectively translocates enzymes to nucleus, which are found in NETs, suggesting that ethanol fixation mimics the process of NETosis. Indeed, direct comparison of ethanol-fixed neutrophils and “NETotic neutrophils” showed that on both substrates P-ANCA-positive sera stain the nucleus (Figures 8A,D), whereas C-ANCA-positive sera stained the cytoplasm (Figures 8B,E). Control sera did not stain NETotic neutrophils (Figure 8C) or ethanol-fixed neutrophils (Figure 8F). Taking together, these data show that NETs and NETotic neutrophils provide a physiological substrate for ANCA testing.

## Discussion

Anti-neutrophil cytoplasmic antibodies were first detected in 1959 in sera from patients with chronic inflammatory diseases using IIF with ethanol-fixed unstimulated neutrophils as substrate (8). According to the current international consensus statement, this technique is still the optimal assay for ANCA screening, and if positive, reflex testing for both PR3- and MPO-ANCA is mandatory (19). IIF has a high sensitivity and specificity, when applied by trained personnel in ANCA-reference laboratories. However, the identification of ANCA-staining patterns by IIF is challenging and error prone for less experienced users (7). Variations in the assay components, such as neutrophil preparations and antibodies, further complicate the reliability of the ANCA screening (7).

Neutrophil extracellular traps may offer improvement over ethanol-fixed naïve neutrophils, which require microscopic identification of intracellular staining patterns. NETs provide a large and spread out surface for antigen binding, making the detection easier and less time consuming. As a proof-of-principle, we used NETs as a substrate to discriminate sera from GPA and MPA patients. The convenience of identification was evident from highly concordant results from individual interpretation by non-experienced personnel. However, additional studies using sera from large patient cohorts are required for identifying a diagnostic benefit of NETs and/or NETotic neutrophils compared to the conventional methods for ANCA testing.

A major weakness of the current ANCA-screening method is that the substrate for ANCA diagnosis is not associated with a pathophysiological state or mechanism; instead, it is based on an *in vitro* artifact produced by ethanol fixation. The distinct ANCA-staining patterns are a result of the neutrophil fixation process with ethanol and do not represent the physiological antigen distribution. In naïve cells, both MPO and PR3 are stored in primary granules. Ethanol permeabilizes the granular membrane, which results in the translocation of MPO to the periphery of the nucleus, whereas PR3 remains within the cytoplasm (7). During NETosis, reactive oxygen species generated by the NADPH oxidase facilitate the release of enzymes from cytoplasmic granules (4). Our data suggest that ethanol fixation of unstimulated neutrophils likely mimics NETosis by perforating granular membranes. Ethanol fixation as well as NETosis causes the translocation of granular enzymes into the nucleus and as a consequence P-ANCAs bind to nuclei of both preparations.

Our study appears to be in disagreement with previous reports, which identified PR3 in NETs by mass spectrometry or IIF (12, 13). However, we do not exclude that low amounts of PR3 are present in NETs, which may be detectable by sensitive methods such as mass spectrometry. The finding that PR3 does not translocate to the nucleus during NETosis supports our results (4).

It is poorly understood how C-ANCAs and P-ANCAs contribute to the severity or progression of GPA and MPA, respectively (10). Our study does not address disease mechanisms, however, concurs with previous findings where NETs and NETotic neutrophils have been implicated in the pathophysiology of several autoimmune diseases including AAV (12, 20–22). Autoantibodies against NET components trigger neutrophils to undergo NETosis, prompting tissue damage and autoimmunity in small vessel vasculitis and systemic lupus erythematosus (12, 23, 24). Furthermore, dendritic cells exposed to NETotic neutrophils trigger ANCA autoimmunity in mice (25), and NET debris has been histologically identified in the microvasculature of patients with small vessel vasculitis (26), suggesting a pathogenic nature of NETs in AAV. We identified that ANCAs can be subdivided into autoantibodies against NETs and autoantibodies against the cell body of netting neutrophils. Future studies may address whether targeting NETs or neutrophil bodies triggers distinct functions in autoimmune disease and AAV.

In conclusion, using a pathophysiologically relevant substrate, such as NETs or NETotic neutrophils, could be an important contribution to the diagnostic repertoire in the assessment of AAV and other ANCA-associated diseases.

## Ethics Statement

This study was carried out in accordance with the recommendations of local ethics guidelines and ethics committees. All subjects gave written informed consent in accordance with the Declaration of Helsinki. The protocol was approved by the ethics committee of the University Medical Center Hamburg-Eppendorf and the Medical Faculty of the Technical University Dresden.

## Author Contributions

RP, LH, and NR developed analytical protocols and analyzed patient samples. TR provided funding, designed experiments, and wrote the manuscript. NR, KC, EC, and FH provided patient samples, reagents, and designed the study. RP, TK, EC, and TF designed the study and wrote the manuscript. All authors critically read the manuscript and provided constructive comments to the study.

## Conflict of Interest Statement

Content of this study is part of pending patent applications.
